# Capecitabine Plus Aromatase Inhibitor as First Line Therapy for Hormone Receptor Positive, HER2 Negative Metastatic Breast Cancer

**DOI:** 10.3390/curroncol30070454

**Published:** 2023-06-24

**Authors:** Alberto Alvarado-Miranda, Fernando Ulises Lara-Medina, Wendy R. Muñoz-Montaño, Juan W. Zinser-Sierra, Paula Anel Cabrera Galeana, Cynthia Villarreal Garza, Daniel Sanchez Benitez, Jesús Alberto Limón Rodríguez, Claudia Haydee Arce Salinas, Alberto Guijosa, Oscar Arrieta

**Affiliations:** 1 Breast Tumors Unit, Instituto Nacional de Cancerología, Mexico City 14080, Mexico; 2 Gastrointestinal Oncology Unit, Instituto Nacional de Cancerología, Mexico City 14080, Mexico; 3 Breast Cancer Center, Hospital Zambrano Hellion TecSalud, Tecnológico de Monterrey, San Pedro Garza García 66278, Mexico; cynthia.villarreal@tecsalud.mx; 4 Grupo Opción Oncología, Monterrey 64060, Mexico; 5 School of Medicine, Universidad Panamericana, Mexico City 03920, Mexico; 6 Thoracic Oncology Unit, Instituto Nacional de Cancerología (INCan), Mexico City 14080, Mexico

**Keywords:** aromatase inhibitor, capecitabine, metastatic breast cancer, metronomic chemotherapy, combined therapy

## Abstract

(1) Background: recent evidence suggests that long low-dose capecitabine regimens have a synergistic effect with endocrine therapy as aromatase inhibitors (AIs), and might increase overall survival for hormone-receptor-positive, HER2-negative, metastatic breast cancer compared to both treatments. We performed a retrospective study to confirm the efficacy and expand the safety data for capecitabine plus AI (a combination henceforth named XELIA) for this indication. (2) We conducted a single-center retrospective cohort study of 163 hormone receptor-positive metastatic breast cancer patients who received either the XELIA regimen, capecitabine, or an aromatase inhibitor (AI) as single agents in first-line treatment. The primary endpoint was progression-free survival, and the secondary endpoints were overall survival, best objective response, and toxicity incidence. (3) Results: the median progression-free survival for patients receiving XELIA, AI, and capecitabine was 29.37 months (20.91 to 37.84; 95% CI), 20.04 months (7.29 to 32.80; 95% CI) and 10.48 (8.69 to 12.28; 95% CI), respectively. The overall response rate was higher in the XELIA group (29.5%) than in the AI (14.3%) and capecitabine (9.1%) groups. However, the differences in overall survival were not statistically significant. Apart from hand–foot syndrome, there were no statistically significant differences in adverse events between the groups. (4) Conclusions: this retrospective study suggests that progression-free survival and overall response rates improved with the XELIA regimen compared to use of aromatase inhibitors and capecitabine alone. Combined use demonstrated an adequate safety profile and might represent an advantageous treatment in places where CDK 4/6 is not available. Larger studies and randomized clinical trials are required to confirm the effects shown in our study.

## 1. Introduction

Breast cancer is the most common malignant neoplasm among females worldwide [1]. Among all subtypes, hormone receptor-positive (HR+) breast cancer is the most frequent, accounting for 79.8% of the cases. According to Leone et al., after an extensive analysis of the Surveillance, Epidemiology, and End Results (SEER) [2], this subtype tends to have better survival than the others [3,4].

Endocrine therapy (ET), including antiestrogens and aromatase inhibitors (AIs), is the mainstay systemic treatment for HR+/HER2- metastatic breast cancer (mBC), given its favorable therapeutic profile [5,6]. The addition of CDK4/6 inhibitors, instead of chemotherapy, proved to be effective for this group of patients [7,8].

Combining endocrine therapy and chemotherapy is not considered an acceptable treatment, as evidence showing that it improves survival is scarce [9]. Additionally, concomitant chemotherapy and tamoxifen increases the risk of embolic events and related deaths [10]. However, some clinical trials showed that tamoxifen and 5-fluorouracil (5-FU) have a synergistic effect [11,12,13]. Other studies, such as that conducted by Kurebavashi, J. et al., showed that the mentioned combination has an additive effect, inhibiting estrogen receptor positive (ER+) breast cancer cells growth [14].

Recently, a therapeutic protocol focusing on a long low-dose chemotherapy administration regimen, known as metronomic chemotherapy (MCh), was studied. It is an attractive modality due to its clinical response, tolerance, and cost-effectiveness [15,16,17,18]. MCh was previously used successfully in multiple studies of an increasing variety of neoplasms, being a feasible alternative in places where new-generation drugs are not available or in patients with acquired resistance to conventional therapies [18,19,20,21]. This therapeutic regimen promotes tumor-activated endothelial cell apoptosis through the inhibition of pro-angiogenic factor expression, increased cellular senescence, and immune modulation [22,23,24,25].

Metronomic chemotherapy can also be used in combination with ET for chemoendocrine treatment. MCh was recently shown to be an effective alternative to overcome endocrine therapy resistance in patients with breast cancer [19,26]. 

Capecitabine is a cytotoxic oral drug with an adequate safety profile and strong evidence of effectiveness in MCh studies [27]. It was proven to be effective in combination with AI, showing favorable outcomes in several clinical trials [28,29,30,31,32,33]. In 2015, Shankar et al. reported median progression free survival (mPFS) of 21 months for chemoendocrine therapy, 15 months for AI, and 8 months for capecitabine alone [28]. Moreover, a recent phase II clinical trial showed that metronomic capecitabine combined with AIs had good efficacy, minimal toxicity, and good tolerance [31]. 

Based on the data is described in literature, we performed a retrospective study to confirm the efficacy and expand safety evidence for capecitabine and AI, a combination that we henceforth refer to as “XELIA”. In our study, which is first of its kind in Latin-America, we explore the clinical outcomes and toxicity profile in HR-positive, HER2-negative metastatic breast cancer subjected to first-line treatment with either metronomic capecitabine and AIs (XELIA) or either conventional capecitabine or AI.

## 2. Materials and Methods

### 2.1. Study Population

We conducted a single-center retrospective study, including patients diagnosed with mBC in a third-level oncology center, between 1 January 2005 and 31 October 2018. Eligibility criteria included being aged ≥18 years, histologically confirmed estrogen receptor (ER) and/or progesterone receptor (PgR) positive, HER2 negative metastatic breast cancer with no prior treatment for metastatic breast cancer, and adequate hematological, renal, and hepatic function. This study was approved by the Institutional Review Board (IRB). Written informed consent from patients was waived due to the retrospective nature of this study. 

### 2.2. Treatment and Evaluation

Treatment was administered according to the physician’s decision under the financial limitation that precluded the administration of CDK4/6 inhibitors. Patients received either capecitabine in combination with an aromatase inhibitor (capecitabine 650 mg/m^2^ BD between day 1 and day 21 every 28 days, as well as either letrozole 2.5 mg QD, exemestane 25 mg QD, or anastrozole 1 mg QD), single-agent capecitabine (1000 to 1200 mg/m^2^ on days 1 to 14 every 21 days), or single-agent AIs (letrozole 2.5 mg QD, exemestane 25 mg QD, or anastrozole 1 mg QD).

Imaging (PET-CT, CT, MRI, and/or bone scans) was performed at baseline every 13–14 weeks. Body imaging studies were performed until disease progression or the initiation of new anticancer therapy, whichever event occurred first. Responses were categorized as complete response (CR), partial response (PR), stable disease (SD), and progressive disease (PD) according to RECIST v1.1.

Before each treatment course, adverse events were assessed and graded according to the Common Toxicity Criteria of the National Cancer Institute v5.0 (CTCAE v5.0). Hematological and non-hematological toxicity resolution (other than fatigue) was required before the start of each cycle. When the toxicities did not resolve, chemotherapy was delayed for one week, and the patient was re-evaluated. Chemotherapy was discontinued in cases of disease progression or major/unacceptable toxicities.

The primary endpoints of this study were PFS (time elapsed from treatment initiation to progression) and death from any cause. The secondary endpoints were OS (time elapsed from the date of treatment initiation to the date of death or last follow-up visit), best objective response, and toxicity.

### 2.3. Statistical Analysis

Clinicopathological parameters were assessed between the three groups using the chi-square test (χ^2^). The median PFS and OS were calculated using the Kaplan–Meier method. All *p*-values were two-tailed, and a value of ≤0.05 was considered significant. Multivariate analyses were performed using the Cox regression model for PFS and OS to identify independent factors and adjust for baseline characteristics using SPSS for Windows v24.0 (IBM Corp., Armonk, NY, USA).

## 3. Results

### 3.1. Patient Characteristics and Treatment

We included 163 patients, of whom 95 received XELIA, 35 received single-agent AI, and 33 received single-agent capecitabine. The patients’ baseline characteristics are described in Table 1. Except for PgR positivity, no significant differences were observed among the three groups. 

The mean age at breast cancer diagnosis was 53.0 years (23–89), while the mean age at metastasis was 56.7 years (23–91). Of the patients, 77.3% were post-menopausal, 83.4% had an ECOG PS of 0–1, 45.4% had visceral metastasis, and 80.4% were PgR-positive (with an even higher proportion of 97.1% in the AI single-agent hormone therapy group).

The median follow-up time was 37 months, with follow-up times ranging from 4–153 months. The mean duration of first-line treatment was 20.46 months, and the median duration was 13.86. The mean of cycles administered was 34 cycles in the XELIA group (P25:15.9 an P75:70.0), 3.3 cycles in the capecitabine group (P25: 6.4 y P75: 24.1), and 19.87 cycles in the AI group (P25: 8.14 and P75:31.0).

### 3.2. PFS, OS and Best Objective Response

During the follow-up period, 77 patients died; of these patients, 45 were (47.4%) in the combination group, 15 were (42.9%) in the AI group, and 17 were (51.5%) in the capecitabine group. The median PFS for was 20.92 months (CI:95%, 15.01–26.85).

**Figure 1:**
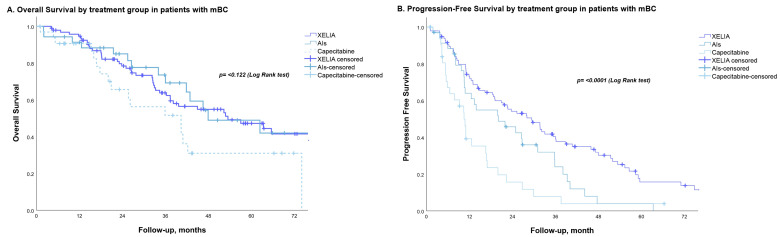
(**A**) Overall survival in patients with metastatic breast cancer receiving first-line treatment with either XELIA, hormone therapy, or chemotherapy. (**B**) Progression-free survival in patients with metastatic breast cancer receiving first-line treatment with either XELIA, hormone therapy, or chemotherapy.

Apart from the number of metastases, no other factors were significantly associated with OS in univariate analysis. No variables were associated with OS in multivariate analysis (Table 2). The capecitabine group had the shortest OS (40.3 months), while the AI and XELIA groups had the longest (54.7 months and 55.1 months, respectively), with no significant differences (*p* = 0.122; Figure 1A).

The median PFS in the AI group was significantly shorter than that in the combination group (20.04 months vs. 29.37 months), and the capecitabine group had the worst prognosis, i.e., 10.48 months (*p* ≤ 0.001; Figure 1B). In univariate analysis, disease state, disease-free interval, the number of metastases, and disease site were also significantly associated with PFS (Table 3).

**Figure 2:**
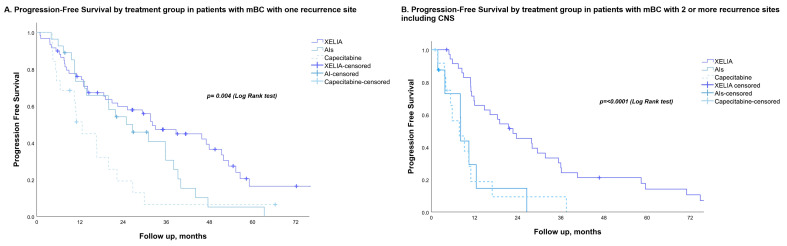
(**A**) Progression-free survival in patients with one-site metastatic breast cancer receiving first-line treatment with either XELIA, hormone therapy, or chemotherapy. (**B**) Progression-free survival in patients with two or more metastatic breast cancers receiving first-line treatment with either XELIA, hormone therapy, or chemotherapy.

**Figure 3:**
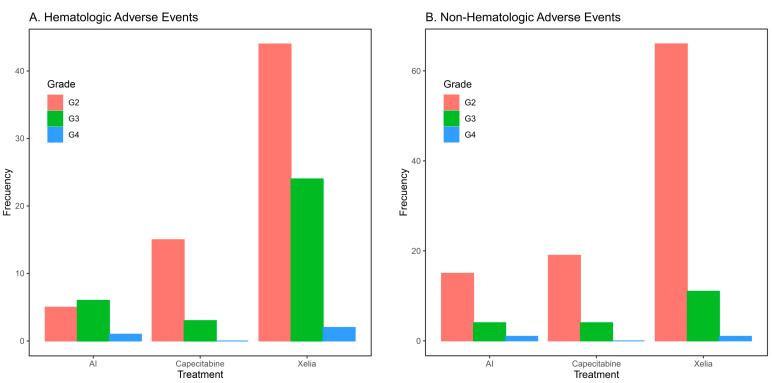
(**A**) Hematological Adverse Event were graded G2–G4. (**B**) Non-Hematological Adverse Events graded G2–G4 frequency of Adverse Event by treatment group is represented in both graphics.

Multivariate analysis showed that XELIA treatment was an independent factor associated with a longer PFS (*p* < 0.001; Table 3). When stratified based on the number of metastases (one vs. two or more; Figure 2) and hormonal status (pre-menopausal vs. post-menopausal; Figure 3), the XELIA group consistently showed a longer PFS, reaching statistical significance.

Table 4 lists the best objective response rates across treatment groups. Both complete and partial responses were higher in the XELIA group. Treatment with XELIA significantly improved the ORR compared to AIs or capecitabine alone (29.5% vs. 14.3% and 9.1%, respectively; *p* = 0.024).

### 3.3. Efficacy and Safety Assessments

Combination therapy (XELIA) was generally well-tolerated. The incidences of adverse events in the treatment group are shown in Table S1. There were no statistically significant differences in toxicity incidences between the groups, except for hand–foot syndrome (palmar–plantar erythrodysesthesia) (*p* = 0.002). A total of 33 patients (34.7%) in the XELIA group had palmar–plantar erythrodysesthesia versus 0% in the AI group. However, this toxicity was most often grade 1–2, with only 3% of the patients presenting with grade 3 toxicity. Hematological and non-hematological adverse events from G2 to G4 (CTCAE v5.0) are represented by frequency in every treatment in Figure 3.

The most common grade 3–4 toxicity was lymphopenia, followed by neutropenia. There were no thromboembolic events in any group. None of the patients permanently discontinued treatment due to adverse events.

## 4. Discussion

The survival of patients with metastatic breast cancer varies from a few months to many years. Hormone receptor status directly correlates with survival. Accordingly, endocrine therapy became one of the major achievements in the management of hormone-sensitive mBC, becoming the current mainstay of treatment and enhancing clinical outcomes and Quality of Life in comparison to chemotherapy [34]. In the past few years, efforts were made to improve patient outcomes and overcome resistance [35].

In recent years, combining AI with a CDK4/6 inhibitor gained importance in the treatment of hormone receptor-positive advanced breast cancer. This combination consistently improved PFS compared to AIs alone [36,37]. Along with these results, other approaches aimed at improving outcomes were previously developed. Two of these strategies were supported by the present study. The first strategy is metronomic chemotherapy, and the second strategy is hormone blockade concomitant with the administration of chemotherapy.

Our single-institution retrospective study showed the efficacy and safety of combining capecitabine with an aromatase inhibitor (letrozole, anastrozole, or exemestane) as a first-line treatment for women with HR-positive metastatic breast cancer. The median progression-free survival of 20.04 months in the aromatase inhibitor group was similar to or greater than that observed in other recent studies of aromatase inhibitors [36,38]. This result may be due to a selection bias, as patients from our study were receiving first-line treatment and had a lower tumor burden; most patients (77.1%) had one metastatic site and, overall, had a lower degree of visceral involvement.

Moreover, XELIA represented a significant improvement in both OS (55.1 vs. 40.3 months) and PFS (29.37 vs. 10.48 months) compared to capecitabine alone. Although there was no difference in the median OS between the XELIA and AI groups, and the secondary endpoint was not met, our results showed that XELIA significantly improved PFS. Disease progression is associated with HRQoL in patients with different metastatic cancers [39]. Thus, PFS improvement in the XELIA group may be related to HRQoL improvement. Importantly, this trend was observed in both pre- and post-menopausal patients, while previous reports focused mainly on the latter group [31]. 

We found an improvement in the overall response rate for patients receiving XELIA (29.5%) compared to AI (14.3%) and capecitabine (9.1%). This difference was statistically significant (*p* = 0.024). The higher rate of complete and partial responses further supports the previously reported synergistic tumoricidal effects of metronomic chemotherapy and hormonal therapy [26].

The recent concern regarding the cost-effectiveness of CDK 4/6 inhibitors [40], along with the results of our study, suggests that utilizing the XELIA regimen might be a feasible alternative. This alternative may be particularly valuable in low-resource settings, where the availability of CDK 4/6 inhibitors is limited.

Regarding safety, our results showed that the combination of metronomic capecitabine with AI is a well-tolerated therapy. It was consistently shown that capecitabine metronomic regimens may be as effective as standard regimens with lower toxicity [16]. Differences in toxicity across groups were minimal, and patients on XELIA seldom developed grades 3–4, which is consistent with prior reports of metronomic capecitabine combined with an aromatase inhibitor [31,41]. It should be noted that neither the present nor previous studies were associated with thromboembolic events.

Although our study had positive results, it had some limitations. The retrospective nature of this study and the fact that a single-center sample was used might restrict its generalizability. Nonetheless, the study’s encouraging results make the XELIA regimen an appealing option for first-line treatment of patients in limited settings in which CDK4/6 are not available. These outcomes indicate the need for further research in a more controlled setting and with the participation of multiple centers. 

## 5. Conclusions

Overall, our results show that the proposed XELIA regimen (metronomic capecitabine plus aromatase inhibitor) resulted in significantly improved progression-free survival and overall response rates compared to use of an aromatase inhibitor or capecitabine alone in women with HR-positive, HER2-negative metastatic breast cancer. Moreover, this combination was shown to have a good safety profile, with low rates of high-grade adverse events and a toxicity profile similar to that with the use of aromatase inhibitor or capecitabine alone.

The XELIA regimen has advantages over endocrine therapy alone and may even represent a cost-effective alternative to CDK 4/6 inhibitors, which could be particularly useful in low-resource settings. Further studies under more controlled conditions are needed to validate the therapeutic value of this regimen before its implementation in clinical practice.

## Figures and Tables

**Table 1:** Baseline characteristics of metastatic breast cancer patients receiving first-line treatment with XELIA, hormone therapy, or chemotherapy. XELIA: capecitabine and aromatase inhibitor; HT: hormone therapy (aromatase inhibitor); CTx: chemotherapy (capecitabine); ECOG PS: Eastern Cooperative Oncology Group Performance Status; SBR: Scarff–Bloom–Richardson; ER: estrogen receptor; PgR: progesterone receptor; IDC: Invasive Ductal Carcinoma; ILC: invasive lobular carcinoma; BCS: breast conservation surgery; MRM: modified radical mastectomy. * regardless of affected site Some patients received both chemotherapy and endocrine therapy as neoadjuvant or adjuvant treatment; ** Visceral involvement included liver, lung, and other visceral metastases. *** Included alone or at any combination sites. Statistically significant *p*-values are shown in bold.

	Total (n = 163)	XELIA (n = 95)	HT (n = 35)	CTx (n = 33)	*p*-Value
**Age, years**					
**Median (range)**	56.00 (23–91)	56.00 (34–87)	56.00 (33–91)	55.00 (23–85) 53.15 (±13.67)	0.958
**Mean (standard deviation)**	56.77 (±12.48)	57.46 (±11.40)	58.29 (±13.79)		
**ECOG PS % (n/N)**					
**0–1**	83.4 (136/163)	84.2 (80/95)	82.9 (29/35)	81.8 (27/33)	0.945
**≥2**	16.6 (27/163)	15.8 (15/95)	17.1 (6/35)	18.2 (6/33)	
**Hormonal status % (n/N)**					
**Pre- or peri-menopausal**	22.7 (37/163)	16.8 (16/95)	28.6 (10/35)	33.3 (11/33)	0.097
**Post-menopausal**	77.3 (126/163)	83.2 (79/95)	71.4 (25/35)	66.7 (22/33)	
**Tumor status % (n/N)**					
**≤T2**	38.0 (62/163)	41.1 (39/95)	40.0 (14/35)	27.3 (9/33)	0.441
**T3**	24.5 (40/163)	22.1 (21/95)	20.0 (7/35)	36.4 (12/33)	
**T4**	37.4 (61/163)	36.8 (35/95)	40.0 (14/35)	36.4 (12/33)	
**Nodal status % (n/N)**					
**N1**	36.2 (59/163)	31.6 (30/95)	45.3 (16/35)	39.4 (13/33)	0.823
**N2**	31.3 (51/163)	32.6 (31/95)	25.7 (8/35)	33.3 (11/33)	
**N3**	17.8 (29/163)	18.9 (18/95)	17.1 (6/35)	15.2 (5/33)	
**Disease state % (n/N)**					
**Recurrent disease**	80.4 (131/163)	78.9 (75/95)	74.3 (26/35)	90.9 (30/33)	0.195
**Metastatic disease**	19.6 (32/163)	21.1 (20/95)	25.7 (9/35)	9.1 (3/33)	
**Histological type % (n/N)**					
**IDC**	82.8 (135/163)	84.2 (80/95)	82.9 (29/35)	78.8 (26/33)	0.776
**ILC**	17.2 (28/163)	15.8 (15/95)	17.1 (6/35)	21.2 (7/33)	
**Ki-67% (n/N)**					
**<20**	47.9 (46/96)	42.9 (24/56)	47.4 (9/19)	61.9 (13/21)	0.329
**≥20**	52.1 (50/96)	57.1 (32/56)	52.6 (10/19)	38.1 (8/21)	
**SBR % (n/N)**					
**Low**	9.1 (13/143)	6.3 (5/80)	18.8 (6/32)	6.5 (2/31)	0.098
**Medium/high**	90.9 (130/143)	93.8 (75/80)	81.3 (26/32)	93.5 (29/31)	
**ER % (n/N)**					
**Negative**	4.3 (7/163)	5.3 (5/95)	5.7 (2/35)	0 (0/33)	0.393
**Positive**	95.7 (156/163)	94.7 (90/95)	94.3 (33/35)	100 (33/33)	
**PgR % (n/N)**					
**Negative**	19.6 (32/163)	21.1 (20/95)	2.9 (1/35)	33.3 (11/33)	**0.006**
**Positive**	80.4 (131/163)	78.9 (75/95)	97.1 (34/35)	66.7 (22/33)	
**ER/PgR % (n/N)**					
**ER+, PgR+**	76.1 (124/163)	73.7 (70/95)	91.4 (32/35)	66.7 (22/33)	**0.021**
**ER+, PgR−**	19.6 (32/163)	21.1 (20/95)	2.9 (1/35)	33.3 (11/33)	
**ER-, PgR+**	4.3 (7/163)	5.3 (5/95)	5.7 (2/35)	0 (0/33)	
**Type of surgery % (n/N)**					
**BCS**	13.0 (17/131)	17.3 (13/75)	7.7 (2/26)	6.7 (2/30)	0.359
**MRM**	85.5 (112/131)	81.3 (61/75)	88.5 (23/26)	93.3 (28/30)	
**Previous neo/adjuvant chemotherapy % (n/N)**					
**Anthracycline-based**	2.3 (3/131)	4.0 (3/75)	0.0 (0/26)	0.0 (0/30)	0.209
**Anthracyclines/taxanes**	86.3 (113/131)	84.0 (63/75)	80.8 (21/26)	96.7 (29/30)	
**Others**	11.5 (15/131)	12.0 (9/75)	19.2 (5/26)	3.3 (1/30)	
**Adjuvant endocrine therapy % (n/N)+**					
**Tamoxifen**	42.7 (56/131)	38.7 (29/75)	50.0 (13/26)	46.7 (14/30)	0.533
**Aromatase inhibitors**	57.3 (75/131)	61.3 (46/75)	50.0 (13/26)	53.3 (16/30)	
**Adjuvant radiotherapy % (n/N)**					
**Yes**	83.2 (109/131)	81.3 (61/75)	84.6 (22/26)	86.7 (26/30)	0.786
**No**	16.8 (22/131)	18.7 (14/75)	15.4 (4/26)	13.3 (4/30)	
**Disease-free interval % (n/N)**					
**≤24 months**	30.5 (40/131)	24.0 (18/75)	34.6 (9/26)	43.3 (13/30)	0.133
**>24 months**	69.5 (91/131)	76.0 (57/75)	65.4 (17/26)	56.7 (17/30)	
**Number of metastases % (n/N)**					
**1 place**	64.4 (105/163)	62.1 (59/95)	77.1 (27/35)	57.6 (19/33)	0.153
**2 places**	22.1 (36/163)	25.3 (24/95)	14.3 (5/35)	21.2 (7/33)	
**≥3 places**	7.4 (12/163)	7.4 (7/95)	8.6 (3/35)	6.1 (2/33)	
CNS involvement *	6.1 (10/163)	5.3 (5/95)	0.0 (0/35)	15.2 (5/33)	
**Disease site % (n/N)**					
Visceral **	45.4 (74/163)	47.4 (45/95)	40.0 (14/35)	45.5 (15/33)	0.138
**Non-visceral**	9.8 (16/163)	11.6 (11/95)	8.6 (3/35)	6.1 (2/33)	
**Bone**	38.7 (63/163)	35.8 (34/95)	51.4 (18/35)	33.3 (11/33)	
CNS ***	6.1 (10/163)	5.3 (5/95)	0.0 (0/35)	15.2 (5/33)	

**Table 2:** Univariate and multivariate analyses of factors associated with overall survival. XELIA: capecitabine and aromatase inhibitor; HT: hormone therapy (aromatase inhibitor); CTx: chemotherapy (capecitabine); ECOG PS: Eastern Cooperative Oncology Group Performance Status; SBR: Scarff–Bloom–Richardson; ER: estrogen receptor; PgR: progesterone receptor; IDC: invasive ductal carcinoma; ILC: invasive lobular carcinoma; BCS: breast conservation surgery; MRM: modified radical mastectomy; X: median not reached; * regardless of affected site. Statistically significant *p*-values are shown in bold.

	Total (Events)	Median (95% CI)	*p*-Value	HR (95% CI)	*p*-Value
ECOG PS			0.972		
0–1	136 (62)	42.9 (31.1–54.7)			
≥2	27 (15)	63.4 (33.9–92.8)			
Hormonal status			0.199		
Pre- or peri-menopausal	54 (19)	46.5 (27.2–65.8)			
Postmenopausal	109 (58)	52.3 (34.5–70.2)			
Tumor status			0.547		
≤T2	62 (25)	62.4 (48.5–76.3)			
T3	40 (21)	40.9 (30.7–51.1)			
T4	61 (31)	41.8 (25.6–58.1)			
Nodal status			0.707		
N0	24 (9)	65.6 (29.7–101.4)			
N1	59 (27)	44.9 (36.7–53.2)			
N2	51 (25)	42.3 (33.4–51.1)			
N3	29 (16)	52.8 (25.7–79.9)			
Disease state			0.947		
Recurrent disease	131 (61)	46.5 (33.1–60.0)			
Metastatic disease	32 (16)	47.9 (16.2–79.7)			
Histological subtype			0.884		
IDC	135 (63)	52.8 (35.5–70.1)			
ILC	28 (14)	37.4 (26.4–48.3)			
Ki67			0.507		
<20	46 (17)	62.39 (27.7–97.1)			
≥20	50 (24)	46.5 (21.7–71.4)			
Unknown	67 (36)	52.3 (32.2–72.5)			
SBR			0.085		
Low	13 (5)	80.1 (31.7–128.6)			
Intermediate and high	130 (66)	40.0 (34.3–47.5)			
Unknown	20 (6)	63.4 (42.4–84.4)			
ER			0.451		
Negative	7 (4)	18.1 (17.8–18.5)			
Positive	156 (73)	47.9 (40.1–64.5)			
PgR			0.447		
Negative	32 (17)	34.3 (11.5–57.3)			
Positive	131 (60)	47.9 (28.1–67.8)			
ER/PgR			0.533		
ER+, PgR+	124 (56)	52.3 (32.6–72.1)			
ER+, PgR−	32 (17)	34.4 (11.5–57.3)			
ER−, PgR+	7 (4)	18.1 (17.8–18.5)			
Type of surgery			0.387		
BCS	17 (5)	98.9 (8.5–189.4)			
MRM	121 (61)	44.9 (34.7–55.2)			
None	25 (11)	36.3 (0–79.57)			
Adjuvant endocrine Therapy			0.748		
Tamoxifen	57 (26)	52.3 (29.6–75.1)			
Aromatase inhibitors	106 (51)	42.8 (26.9–58.8)			
Disease-free interval % (n/N)			0.993		
Newly metastatic disease	32 (16)	47.9 (16.6–79.7)			
≤24 months	40 (18)	46.5 (24.3–68.7)			
>24 months	91 (43)	44.9 (30.2–59.7)			
Number of metastases % (n/N)			**0.010**	1.129 (0.879–1.449)	0.341
1 place	105 (47)	53.5 (34.9–72.1)			
2 places	36 (22)	34.4 (30.0–47.8)			
≥3 places	12 (6)	26.4 (9.5–43.3)			
CNS involvement *	10 (2)	X			
Disease site % (n/N)			0.068		
Visceral	84 (40)	40.4 (21.4–59.4)			
Non-visceral	16 (3)	X			
Bone	63 (34)	44.4 (37.3–52.6)			
Treatment			0.122	1.262 (0.947–1.683)	0.112
XELIA	95 (45)	53.5 (30.3–76.6)			
Hormone therapy	35 (15)	47.9 (24.6–71.2)			
Chemotherapy	33 (17)	40.4 (34.7–61.3)			

**Table 3:** Univariate and multivariate analyses of factors associated with progression-free survival. XELIA: Capecitabine and Aromatase Inhibitor; HT: hormone therapy (aromatase inhibitor); CTx: chemotherapy (capecitabine); ECOG PS: Eastern Cooperative Oncology Group Performance Status; SBR: Scarff–Bloom–Richardson; ER: estrogen receptor; PgR: progesterone receptor; IDC: invasive ductal carcinoma; ILC: invasive lobular carcinoma; BCS: breast conservation surgery; MRM: modified radical mastectomy; * regardless of affected site. Statistically significant *p*-values are shown in bold.

	Total (Events)	Median	*p*-Value	HR (95% CI)	*p*-Value
		(95% CI)			
ECOG PS					
0–1	136 (108)	18.7 (12.3–25.1)	0.215		
≥2	27 (19)	36.1 (16.9–55.4)			
Hormonal status					
Pre- or peri-menopausal	54 (44)	16.3 (8.0–24.6)	0.082		
Post-menopausal	109 (83)	22.5 (14.6–30.5)			
Tumor status					
≤T2	62 (44)	28.0 (16.9–39.2)	0.134		
T3	40 (34)	18.7 (12.6–24.7)			
T4	61 (49)	19.0 (10.5–27.6)			
Nodal status					
N0	24 (16)	27.9 (13.0–42.7)	0.184		
N1	59 (45)	24.9 (18.5–31.3)			
N2	51 (43)	12.9 (7.2–18.7)			
N3	29 (23)	16.9 (5.9–27.9)			
Disease state					
Recurrent disease	131 (104)	16.7 (10.2–23.2)	**0.039**	0.805 (0.507–1.281)	0.361
Metastatic disease	32 (23)	31.1 (20.9–41.2)			
Histological subtype					
IDC	135 (104)	20.9 (14.0–27.8)	0.992		
ILC	28 (23)	20.0 (8.1–32.0)			
Ki67					
<20	46 (35)	23.7 (10.1–37.2)	0.428		
≥20	50 (36)	12.9 (4.7–20.2)			
SBR					
Low	13 (11)	26.7 (15.3–38.0)	0.731		
Intermediate and high	130 (102)	20.0 (14.9–25.1)			
ER					
Negative	7 (5)	12.9 (0.1–25.8)	0.952		
Positive	156 (122)	20.9 (14.9–27.2)			
PgR					
Negative	32 (21)	19.1 (12.2–26.0)	0.998		
Positive	131 (106)	20.9 (13.9–27.9)			
ER/PgR					
ER+, PgR+	124 (101)	20.9 (13.5–28.4)	0.729		
ER+, PgR−	32 (21)	19.1 (12.2–26.0)			
ER−, PgR+	7 (5)	12.9 (0.1–25.8)			
Type of surgery					
BCS	17 (11)	22.3 (0.0–60.60)	0.731		
MRM	112 (92)	16.6 (10.9–22.2)			
Adjuvant endocrine therapy					
Tamoxifen	56 (48)	19.9 (9.1–30.6)	0.776		
Aromatase inhibitors	75 (56)	16.7 (10.7–22.7)			
Disease-free interval % (n/N)					
Newly metastatic disease	32 (23)	31.0 (20.9–41.2)	**0.016**	1.069 (0.690–1.657)	0.765
≤24 months	40 (30)	10.5 (6.7–14.3)			
>24 months	91 (74)	19.9 (13.8–26.1)			
Number of metastases % (n/N)					
1 place	105 (78)	25.2 (16.4–34.1)	**0.016**	1.494 (1.051–2.125)	**0.025**
2 places	36 (32)	16.3 (8.9–23.7)			
≥3 places	12 (12)	8.5 (4.6–12.4)			
CNS involvement *	10 (5)	19.0 (0.0–60.9)			
Disease site % (n/N)					
Visceral	84 (69)	13.9 (9.4–18.3)	**0.037**	1.430 (1.070–1.912)	**0.016**
Non-visceral	16 (10)	11.0 (0–49.7)			
Bone	63 (48)	29.4 (20.2–38.6)			
Treatment					
XELIA	95 (70)	29.37 (20.91–37.84)	**<0.001**	1.669 (1.330–2.094)	**<0.001**
Hormone therapy	35(30)	20.04 (7.29–32.79)			

**Table 4:** Best objective response rate by treatment group. XELIA, capecitabine and aromatase inhibitor; HT: hormone therapy (aromatase inhibitor); CTx: chemotherapy (capecitabine); PR: partial response; CR: complete response; SD: stable disease. Statistically significant *p*-values are shown in bold.

Best Objective Response	XELIA (n = 95) n (%)	HT (n = 35) n (%)	CTx (n = 33) n (%)	*p*-Value
Complete response	10 (10.5)	2 (5.7)	0 (0.0)	0.125
Partial response	18 (18.9)	3 (8.6)	3 (9.1)	0.198
Stable disease	45 (47.4)	18 (51.4)	22 (66.7)	0.160
Progressive disease	22 (23.2)	12 (34.3)	8 (24.2)	0.426
Overall response rate (PR + CR)	28 (29.5)	5 (14.3)	3 (9.1)	**0.024**
Disease control rate (PR + CR + SD)	73 (76.8)	23 (65.7)	25 (75.8)	0.426

## Data Availability

Not applicable.

## Supplementary Materials

The following supporting information can be downloaded at: https://www.mdpi.com/article/10.3390/curroncol30070454/s1, Table S1: Toxic Effects by treatment group in patients with metastatic breast cancer patients.
