# Correction: Alvarado-Miranda et al. Capecitabine Plus Aromatase Inhibitor as First Line Therapy for Hormone Receptor Positive, HER2 Negative Metastatic Breast Cancer. *Curr. Oncol.* 2023, *30*, 6097–6110

**DOI:** 10.3390/curroncol31110529

**Published:** 2024-11-15

**Authors:** Alberto Alvarado-Miranda, Fernando Ulises Lara-Medina, Wendy R. Muñoz-Montaño, Juan W. Zinser-Sierra, Paula Anel Cabrera Galeana, Cynthia Villarreal Garza, Daniel Sanchez Benitez, Jesús Alberto Limón Rodríguez, Claudia Haydee Arce Salinas, Alberto Guijosa, Oscar Arrieta

**Affiliations:** 1 Breast Tumors Unit, Instituto Nacional de Cancerología, Mexico City 14080, Mexico; 2 Gastrointestinal Oncology Unit, Instituto Nacional de Cancerología, Mexico City 14080, Mexico; 3 Breast Cancer Center, Hospital Zambrano Hellion TecSalud, Tecnológico de Monterrey, San Pedro Garza García 66278, Mexico; cynthia.villarreal@tecsalud.mx; 4 Grupo Opción Oncología, Monterrey 64060, Mexico; 5 School of Medicine, Universidad Panamericana, Mexico City 03920, Mexico; 6 Thoracic Oncology Unit, Instituto Nacional de Cancerología (INCan), Mexico City 14080, Mexico

In the original publication [1], there was a mistake in Table 2 as published. The original Table 2 considered different amounts of patients for the survival analysis at the data cut-off. The corrected Table 2 appears below. The authors state that the scientific conclusions are unaffected. This correction was approved by the Academic Editor. The original publication has also been updated.

## Figures and Tables

**Table 2:** Univariate and multivariate analyses of factors associated with overall survival. XELIA: capecitabine and aromatase inhibitor; HT: hormone therapy (aromatase inhibitor); CTx: chemotherapy (capecitabine); ECOG PS: Eastern Cooperative Oncology Group Performance Status; SBR: Scarff–Bloom–Richardson; ER: estrogen receptor; PgR: progesterone receptor; IDC: invasive ductal carcinoma; ILC: invasive lobular carcinoma; BCS: breast conservation surgery; MRM: modified radical mastectomy; X: median not reached; * regardless of affected site. Statistically significant *p*-values are shown in bold.

	Total (Events)	Median (95% CI)	*p*-Value	HR (95% CI)	*p*-Value
ECOG PS			0.972		
0–1	136 (62)	42.9 (31.1–54.7)			
≥2	27 (15)	63.4 (33.9–92.8)			
Hormonal status			0.199		
Pre- or peri-menopausal	54 (19)	46.5 (27.2–65.8)			
Postmenopausal	109 (58)	52.3 (34.5–70.2)			
Tumor status			0.547		
≤T2	62 (25)	62.4 (48.5–76.3)			
T3	40 (21)	40.9 (30.7–51.1)			
T4	61 (31)	41.8 (25.6–58.1)			
Nodal status			0.707		
N0	24 (9)	65.6 (29.7–101.4)			
N1	59 (27)	44.9 (36.7–53.2)			
N2	51 (25)	42.3 (33.4–51.1)			
N3	29 (16)	52.8 (25.7–79.9)			
Disease state			0.947		
Recurrent disease	131 (61)	46.5 (33.1–60.0)			
Metastatic disease	32 (16)	47.9 (16.2–79.7)			
Histological subtype			0.884		
IDC	135 (63)	52.8 (35.5–70.1)			
ILC	28 (14)	37.4 (26.4–48.3)			
Ki67			0.507		
<20	46 (17)	62.39 (27.7–97.1)			
≥20	50 (24)	46.5 (21.7–71.4)			
Unknown	67 (36)	52.3 (32.2–72.5)			
SBR			0.085		
Low	13 (5)	80.1 (31.7–128.6)			
Intermediate and high	130 (66)	40.0 (34.3–47.5)			
Unknown	20 (6)	63.4 (42.4–84.4)			
ER			0.451		
Negative	7 (4)	18.1 (17.8–18.5)			
Positive	156 (73)	47.9 (40.1–64.5)			
PgR			0.447		
Negative	32 (17)	34.3 (11.5–57.3)			
Positive	131 (60)	47.9 (28.1–67.8)			
ER/PgR			0.533		
ER+, PgR+	124 (56)	52.3 (32.6–72.1)			
ER+, PgR−	32 (17)	34.4 (11.5–57.3)			
ER−, PgR+	7 (4)	18.1 (17.8–18.5)			
Type of surgery			0.387		
BCS	17 (5)	98.9 (8.5–189.4)			
MRM	121 (61)	44.9 (34.7–55.2)			
None	25 (11)	36.3 (0–79.57)			
Adjuvant endocrine Therapy			0.748		
Tamoxifen	57 (26)	52.3 (29.6–75.1)			
Aromatase inhibitors	106 (51)	42.8 (26.9–58.8)			
Disease-free interval % (n/N)			0.993		
Newly metastatic disease	32 (16)	47.9 (16.6–79.7)			
≤24 months	40 (18)	46.5 (24.3–68.7)			
>24 months	91 (43)	44.9 (30.2–59.7)			
Number of metastases % (n/N)			**0.010**	1.129 (0.879–1.449)	0.341
1 place	105 (47)	53.5 (34.9–72.1)			
2 places	36 (22)	34.4 (30.0–47.8)			
≥3 places	12 (6)	26.4 (9.5–43.3)			
CNS involvement *	10 (2)	X			
Disease site % (n/N)			0.068		
Visceral	84 (40)	40.4 (21.4–59.4)			
Non-visceral	16 (3)	X			
Bone	63 (34)	44.4 (37.3–52.6)			
Treatment			0.122	1.262 (0.947–1.683)	0.112
XELIA	95 (45)	53.5 (30.3–76.6)			
Hormone therapy	35 (15)	47.9 (24.6–71.2)			
Chemotherapy	33 (17)	40.4 (34.7–61.3)			
