# Supplementary material for: Capecitabine Plus Aromatase Inhibitor as First Line Therapy for Hormone Receptor Positive, HER2 Negative Metastatic Breast Cancer
Source: Curr Oncol. 2023 Jun 24;30(7):0. doi: 10.3390/curroncol30070454 (PMC10377785; doi:10.3390/curroncol30070454)
Supplement: Supplementary file 1 [file curroncol-30-00454-s001.zip › curroncol-2430951-supplementary.pdf]

Table S1. Toxic Effects by treatment group in patients with metastatic breast cancer patients. Grades of toxic effects were defined according to the Common Toxicity Criteria of the National Cancer Institute (version 5.0). XELIA: Capecitabine + Aromatase inhibitor; HT: Hormone Therapy (Aromatase Inhibitor); CTx: Chemotherapy (Capecitabine); AST = aspartate aminotransferase; ALT = alanine aminotransferase.

**Toxicity**	**Grade 1 % (n)**			**Grade 2 % (n)**			**Grade 3 % (n)**			**Grade 4 % (n)**			**Grade 3+4 % (n)**			**All grade toxicities % (n)**			***p*-value**
	**Xelia**	**HT**	**CTx**	**Xelia**	**HT**	**CTx**	**Xelia**	**HT**	**CTx**	**Xelia**	**HT**	**CTx**	**Xelia**	**HT**	**CTx**	**Xelia**	**HT**	**CTx**	
Leukopenia	45.3(43)	57.1(20)	63.6(21)	10.5(10)	0(0)	3.0(1)	2.1 (2)	2.9(1)	3.0(1)	0 (0)	0(0)	0(0)	2.1 (2)	2.9(1)	3.0(1)	57.9(55)	60.0(21)	59.7(24)	0.245
Neutropenia	3.2(3)	2.9 (1)	3.0 (1)	10.5(10)	2.9 (1)	3.0 (1)	10.5(10)	0(0)	0(0)	0(0)	0(0)	0(0)	10.5(10)	0(0)	0(0)	16.0(15)	5.8(2)	6.0(2)	0.547
Lymphopenia	45.3(43)	42.9(15)	36.4(12)	16.8(16)	8.6(3)	30.3(10)	9.5(9)	8.6(3)	3.0(1)	1.1(1)	0(0)	0(0)	10.6(10)	8.6(3)	3.0(1)	72.6(69)	60.0(21)	69.7(23)	0.394
Anemia	17.9(17)	8.6(3)	27.3(9)	6.3(6)	0(0)	0(0)	2.1(2)	2.9(1)	3.0(1)	0(0)	0(0)	0(0)	2.1(2)	2.9(1)	3.0(1)	26.3(30)	11.4(4)	30.3(10)	0.190
Thrombocytopenia	17.9(17)	14.3(5)	15.2(5)	2.1(2)	2.9(1)	9.1(3)	1.1(1)	2.9(1)	0(0)	1.1(1)	2.9(1)	0(0)	2.1(2)	5.8(2)	0(0)	22.1(26)	22.9(8)	24.3(8)	0.651
Creatinine elevation	3.2(3)	2.9(1)	3.0(1)	3.2(3)	2.9(1)	0(0)	2.1(2)	0(0)	0(0)	1.1(1)	2.9(1)	0(0)	3.2(3)	2.9(1)	0(0)	9.5(9)	8.6(3)	3.0(1)	0.878
AST elevation	26.3(25)	31.4(11)	18.2(6)	2.1(2)	0(0)	9.1(3)	1.1(1)	0(0)	0(0)	0(0)	0(0)	0(0)	1.1(1)	0(0)	0(0)	29.5(28)	31.4(11)	27.3(9)	0.302
ALT elevation	7.4(7)	20.0(7)	12.1(4)	2.1(2)	0(0)	3.0(1)	0(0)	0(0)	0(0)	0(0)	0(0)	0(0)	0(0)	0(0)	0(0)	9.5(9)	20.0(7)	15.1(5)	0.285
Triglycerides	6.3(6)	5.7(2)	0(0)	7.4(7)	5.7(2)	0(0)	2.1(2)	2.9(1)	3.0(1)	0(0)	0(0)	0(0)	2.1(2)	2.9(1)	3.0(1)	15.8(15)	14.3(5)	3.0(1)	0.538
Albumin	43.2(41)	37.1(13)	42.4(14)	12.6(12)	14.3(5)	9.1(3)	2.1(2)	5.7(2)	6.1(2)	0(0)	0(0)	0(0)	2.1(2)	5.7(2)	6.1(2)	57.9(55)	57.1(20)	57.6(19)	0.903
Bilirubin	10.5(10)	0(0)	9.1(3)	5.3(5)	5.7(2)	0(0)	0(0)	2.9(1)	0(0)	0(0)	0(0)	0(0)	0(0)	2.9(1)	0(0)	15.8(15)	8.6(3)	9.1(3)	0.147
Hand / Foot syndrome	12.6(12)	0(0)	21.2(7)	18.9(18)	0(0)	21.2(7)	3.2(3)	0(0)	3(1)	0(0)	0(0)	0(0)	3.2(3)	0(0)	3(1)	34.7(33)	0(0)	45.5(15)	***0.002***
Diarrhea	8.4(8)	5.7(2)	12.1(4)	9.5 (9)	0(0)	6.1(2)	0(0)	0(0)	0(0)	0(0)	0(0)	0(0)	0(0)	0(0)	0(0)	17.9(17)	5.7(2)	18.2(6)	0.315
Nausea/Vomiting	31.6(30)	17.1(6)	39.4(13)	7.4(7)	11.4(4)	9.1(3)	1.1(1)	0(0)	0(0)	0(0)	0(0)	0(0)	1.1(1)	0(0)	0(0)	40.0(48)	28.6(10)	48.5(16)	0.503
Mucositis	3.2(3)	0(0)	0(0)	1.1(1)	0(0)	0(0)	0(0)	0(0)	0(0)	0(0)	0(0)	0(0)	0(0)	0(0)	0(0)	4.3(4)	0(0)	0(0)	0.569
